# Identifying barriers for out of hospital emergency care in low and low-middle income countries: a systematic review

**DOI:** 10.1186/s12913-018-3091-0

**Published:** 2018-04-19

**Authors:** Antony Gatebe Kironji, Peter Hodkinson, Sarah Stewart de Ramirez, Trisha Anest, Lee Wallis, Junaid Razzak, Alexander Jenson, Bhakti Hansoti

**Affiliations:** 1 Johns Hopkins School of Medicine, Baltimore, MD USA; 2 Division of Emergency Medicine, University of Cape Town, Cape Town, South Africa; 3 Department of Emergency Medicine, Johns Hopkins Hospital, Baltimore, MD USA

**Keywords:** Emergency medicine, Barriers to prehospital care, Prehospital care, Out of hospital emergency care, Low-middle income countries (LMIC), Low income countries (LIC)

## Abstract

**Background:**

Out-of-hospital emergency care (OHEC), also known as prehospital care, has been shown to reduce morbidity and mortality from serious illness. We sought to summarize literature for low and low-middle income countries to identify barriers to and key interventions for OHEC delivery.

**Methods:**

We performed a systematic review of the peer reviewed literature from January 2005 to March 2015 in PubMed, Embase, Cochrane, and Web of Science. All articles referencing research from low and low-middle income countries addressing OHEC, emergency medical services, or transport/transfer of patients were included. We identified themes in the literature to form six categories of OHEC barriers. Data were collected using an electronic form and results were aggregated to produce a descriptive summary.

**Results:**

A total 1927 titles were identified, 31 of which met inclusion criteria. Barriers to OHEC were divided into six categories that included: culture/community, infrastructure, communication/coordination, transport, equipment and personnel. Lack of transportation was a common problem, with 55% (17/31) of articles reporting this as a hindrance to OHEC. Ambulances were the most commonly mentioned (71%, 22/31) mode of transporting patients. However, many patients still relied on alternative means of transportation such as hired cars, and animal drawn carts. Sixty-one percent (19/31) of articles identified a lack of skilled personnel as a key barrier, with 32% (10/31) of OHEC being delivered by laypersons without formal training. Forty percent (12/31) of the systems identified in the review described a uniform access phone number for emergency medical service activation.

**Conclusions:**

Policy makers and researchers seeking to improve OHEC in low and low-middle income countries should focus on increasing the availability of transport and trained providers while improving patient access to the OHEC system. The review yielded articles with a primary focus in Africa, highlighting a need for future research in diverse geographic areas.

## Background

The development of emergency care systems is a growing focus in low-middle income countries (LMIC). It is estimated that as many as 45% of deaths and 35% of disability-adjusted life years can be addressed by developing robust emergency care systems in LMIC [1]. One of the biggest challenges in many rural low resource settings is the scarcity of emergency care, and where present, the distance and time to access appropriate services [2–4].

Out of Hospital Emergency Care (OHEC), commonly understood as prehospital care, refers to the acute and emergency care delivered outside the walls of a fixed health facility/hospital [5]. It has previously been demonstrated that in LMIC without formal emergency care systems, nearly 80% of deaths due to severe injury occurred in the prehospital setting [6]. Developing prehospital trauma care systems has been emphasized as an integral component of the healthcare system [7].

OHEC includes a spectrum of care delivery from first responder care (FRC), prehospital care (PHC), and emergency medical services (EMS). OHEC is an umbrella term coined by the African Federation for Emergency Medicine (AFEM) in 2013. It begins with first responder care upon recognition of a perceived or actual emergency and includes the full spectrum of emergency care that occurs outside of healthcare facilities.

Less than 1% of the population in many low-income countries (LIC) has access to formal emergency medical transportation services, such as ambulances [8]. In sub-Saharan Africa and Asia those few who do have access to medical transport would typically only have transport between facilities and not from the scene of injury [9]. Delays in reaching health facilities has been shown to lead to poor patient outcomes [3], yet up to 60% of individuals living in a LMIC live more than 8 km away from the nearest health facility/hospital [10]. Given the scarcity of emergency care facilities in LMIC, [5] OHEC can be crucial in determining patient survival.

The majority of the literature to date in LMIC focuses on barriers for OHEC in specific countries often with disease specific focuses (e.g. trauma or obstetric care) [11–18]. Additionally, the most recent review that describes barriers to emergency care was conducted in 2012 [19]. While this study focuses on barriers to care in both high and low income countries, that study predominantly emphasized financial barriers to care delivery. This review performs a comprehensive systematic search of the peer-reviewed literature across four different databases to identify barriers to and also key interventions for OHEC delivery in LMIC.

## Methods

### Criteria for considering studies in this review

This systematic review included review articles, observational studies, quantitative studies, qualitative studies, evaluations/report articles and policy papers that addressed OHEC. This included articles that addressed (1) emergency care for patients prior to arriving at the hospital (first responder care or prehospital care) and (2) systems in place to respond to health emergencies. All health conditions were included. Studies that did not include LIC or LMIC as defined by the World Bank (http://data.worldbank.org/about/country-and-lending-groups), studies focusing only on care in hospital settings, case based studies (*n* < 5), editorials or descriptive studies were excluded (Table 1).

**Table 1:** Inclusion and exclusion criteria used to screen articles

Inclusion	Exclusion
LIC or LMIC as defined by the World Bank (http://data.worldbank.org/about/country-and-lending-groups).	Non-English language; Full article not found
Study focus: All studies addressing Out of hospital emergency care which includes First responder care, Prehospital care, and Emergency medical care	Study focus: Hospital care
Disease focus: All disease conditions	
Type of study: Observational, Evaluation/reports, Expert review, and Qualitative studies	Type of study: Case based studies (*n* < 5), editorials and descriptive studies

### Search methods for identification of studies

We searched MEDLINE (via PubMed), Embase, Cochrane, and Web of Science to find potential articles from January 2005 to March 2015. We included combined controlled vocabulary words (MeSH) and related keywords for 1) barriers to care, 2) emergency medical care (FRC, PHC, and EMS) and 3) developing country. See Appendix 1: Search Strategy. This search explored database Boolean operators (‘OR’ for related/similar terms and ‘AND’ to combine different concepts) to combine these three key ideas. We consulted an informationist (KL) from Johns Hopkins University Welch Medical Library for each search strategy. Results in languages other than English were excluded.

### Data collection and analysis

#### Selection of studies

We imported all results from the four data bases into Refworks for initial organization of the results. Prior to review, duplicate records were removed using Refworks. For the review, all relevant articles were exported from Refworks into Excel. A team of six researchers (GK, BH, AJ, PH, TA and SSR) were responsible for conducting the entire review process. Two reviewers independently screened each title and abstract. Using the inclusion and exclusion criteria, the reviewers evaluated the titles and abstracts to see if they should receive a full text review marking them as “Yes” or “No”. Articles were marked as “No” only if they violated any of the exclusion criteria. A “Yes” vote was assigned to articles that did not violate the exclusion criteria. All articles marked “Yes” during the title and abstract review were included for the full text review. Any conflict regarding study inclusion were resolved by a third reviewer.

In the title and abstract screening process articles were excluded if they (1) didn’t include at least one LIC or LMIC (2) didn’t address OHEC (3) only included in-hospital emergency care (4) were case based studies (*n* < 5), editorials or descriptive studies (4) none English.

**Fig. 1:**
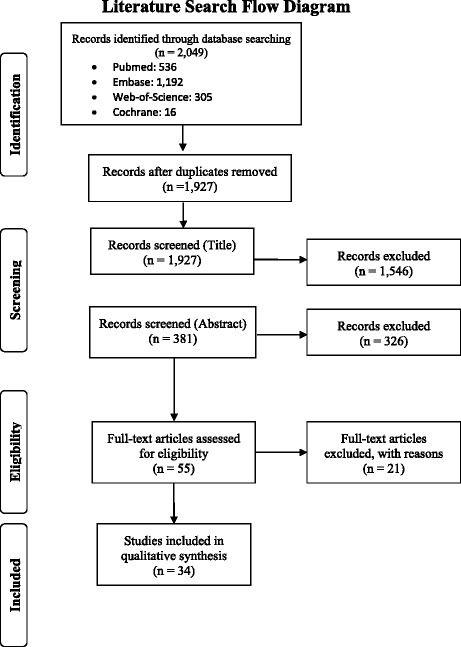
Summary of sources contributing to the systematic review

For the full text review, one reviewer was assigned to each article. All articles were obtained by the study team by searching google, MEDLINE, and the Johns Hopkins Welch Library online resources. The reviewers screened the full text for presences of inclusion criteria and violation of exclusion criteria. The same inclusion and exclusion criteria listed above were applied to the full-text review. After screening the text, reviewers selected to either include or exclude the article into the systematic review. The screening process was conducted in accordance with Preferred Reporting Items for Systematic Reviews and Meta-Analyses (PRISMA) guidelines, Fig. 1 shows the PRISMA process flow diagram.

#### Data extraction and management

Data extraction for the full text was performed by five reviewers (GK, BH, AJ, PH, and TA). Each article was assigned to one reviewer. All reviewers used standardized definitions and examples for the extraction process. Data was collected with an electronic form that utilized multiple choice as well as free text options to capture responses. For each article the review recorded: 1) the study site, 2) information on the OHEC system as described by the article (which included level of first responder training, types of emergency transport available, patient population being served, type of EMS system available, and availability of an emergency contact number), 3) summary of barriers to delivery of OHEC addressed in the article within the following domains: health access, communication, medical equipment, availability and training of first responders, coordination of the EMS system, triage of patients and finances, 4) summary of the EMS pathway where barriers exist (community, dispatch, emergency responders, transportation, and/or roads). See Table 2. For each barrier domain and EMS pathway selected the reviewer provided evidence from the article using quotes or paraphrasing.

**Table 2:** Summary of Reviewed articles BMC. Overview of all articles included in the systematic review

First Author and year published	Country	Study design	Population/ Disease studied	Type of EMS system	Level of training of EMS providers	How the community access the EMS system	EMS transport vehicles	OHEC Barrier Category	Specific issues/barriers raised in the article
Cham et al. 2005 [11]	Gambia	Cross sectional study Interview based qualitative study	General obstetrics	Hospital based dispatch	Not addressed	Walk to central location	Ambulance Personal cars	Culture/community Infrastructure Transport	• Lack of knowledge of danger signs • Unfavorable experience with health system • Roads are in poor condition • Facility inappropriate to treat patient • Transportation not available • Ambulance lacked fuel
Kobusingye et al. 2005 [9]	Multiple countries	Expert review paper Expert review with recommendations	General	N/A	N/A	N/A	N/A	Communication/Coordination Equipment Infrastructure Personnel	• Patient access issues • Communication • Equipment • Coordination and management of EMS system • Skilled personnel • Triage prioritization
Thomson 2005 [2]	Zimbabwe	Cross sectional study Descriptive study of the development of emergency medical services	General	EMS with paramedics dispatched	Layperson First Aid EMT Nurse	Call designated number	Ambulance Ox-drawn ambulance	Communication/Coordination Transport Equipment Personnel	• Dispatcher is not available or has variable level of training • Ambulances travel very long distance to respond to emergencies • Ambulances are not well equipped to respond to emergencies • Providers lacked formal prehospital training
Ali et al. 2006 [35]	Pakistan	Cross sectional study Retrospective data analysis + Interviews of key personnel and survey of community members	General	Physician respond	First Aid Physician	Call designated number	Ambulance	Infrastructure Equipment Personnel	• Challenge with road navigation • Lack of equipment • Providers lacked formal prehospital training
Kawuwa et al. 2007 [12]	Nigeria	Cross sectional study Semi-structured interviews of community representatives	Obstetric complications	None	Layperson	None	Ambulance Other non-ambulance vehicles (bikes, buses, motorbike, etc.)	Culture/Community Infrastructure Transport	• Lack of knowledge of danger signs • Preference of traditional/spiritual therapies • Women rely on husband to seek care • Community perception of care facility influence whether to seek care • Poor road conditions especially during rainy season • Unreliable transportation
Hofman et al. 2008 [13]	Malawi	Evaluation/Report Observational study after new intervention was implemented	Obstetrics	Hospital based dispatch	Community health workers	Walk to central location	Motocycle ambulance, Ambulance Private vehicles, Bicycles, Motorcycle	Infrastructure Communicaton/Coordination Transport	• Poor road conditions especially during rainy season • Lack of means to directly communicate with local hospital • Reliance on alternative means of transportation • Long transport time
Siddiqui et al. 2008 [14]	Pakistan	Cross sectional study Survey conducted of patients with stroke at one hospital	Adult stroke	Not mentioned	Not mentioned	Not mentioned	Ambulance	Culture/Community Infrastructure Transport Personnel	• Lack of knowledge of danger signs • Facility inappropriate to teat patient • Lack of effective ambulance services • Doctors to training to be able refer patients appropriately
Jayaraman et al. 2009 [29]	Uganda	Cross sectional study Survey conducted of prehospital providers: police officers, minibus taxi drivers, and local council officials.	General trauma	No system	Layperson First aid	None	Personal car Taxi cars	Personnel Equipment	• First responders lack training • Lack of first aid equipment
Macharia et al. 2009 [36]	Kenya	Cross sectional study Survey of patients and health personnel at 53 hospitals	General trauma	Not mentioned	Layperson	Not mentioned	Ambulance Personal car	Personnel	• Lack of trained emergency responders
Khan et al. 2010 [33]	Pakistan	Cross sectional study Retrospective analysis of data from one hospital’s trauma database	General trauma	None	Layperson	None	Personal means	Infrastructure Personnel	• Facility inappropriate to treat patient • Lack of trained personnel in the prehospital setting
Mahmood et al. 2010 [32]	Pakistan	Cross sectional study Descriptive study based on observation of patients interaction with EMS	General	EMS with paramedics dispatched	EMT	Call designated number	Ambulance	Infrastructure Personnel Equipment	• Traffic congestion • Emergency responders lack training • Lack of equipment (spine board, burn and airway kit)
Roy et al. 2010 [15]	India	Cross sectional study Semi-structured interview of admitted patients or informants	General trauma	None	Layperson	None	Ambulance Taxi cars Police Vans	Infrastructure Communication/Coordination Transport Equipment Personnel	• Facility inappropriate to treat patient • Lack of coordination of emergency responders • Transport done primarily using non-ambulance vehicle • Long transport distance • Ambulance lack resuscitation equipment • Emergency responders use inappropriate treatment in the prehospital setting
Essendi,et al. 2011 [21]	Kenya	Qualitative Interview based qualitative study	General obstetrics	Not mentioned	Not mentioned	Not mentioned	Not mentioned	Culture/Community Infrastructure Transport	• Lack of knowledge on danger signs • Preference to see TBA over going to health center • Woman rely on husband or other family member to seek care • Poor road condition • Insecurity at night • Facility inappropriate to treat patient • Lack of availability of ambulance • To use ambulance patient must first provide fuel
Jammeh et al. 2011 [3]	Gambia	Cross sectional study Interview based qualitative study	Obstetrics emergencies	Not mentioned	Layperson	None	Donkey Bike	Culture/Community Infrastructure Transport	• Lack of knowledge on danger signs • Women lack autonomy • Poor road conditions especially in rainy season • Transportation mode inconvenient for laboring woman • Long transport time • Lack of availability of ambulance
Wen et al. 2011 [28]	Rwanda	Cross sectional study Descriptive study based on observations and Interviews	General	Not mentioned	Not mentioned	None	Ambulance Personal car Taxi cars Taxi motobikes Hired individual cars	Infrastructure Communication/Coordination Transport Personnel	• Facility inappropriate to treat patient • No prehospital triage • No prehospital care and coordination of services • Few ambulances available • Ambulance used infrequently • Lack of trained providers
Adewole et al. 2012 [22]	Nigeria	Longitudinal study Retrospective analysis of data on ambulance services	General	EMS with paramedics dispatched	First aid Nurse	Call designated number	Ambulance	Culture/Community Infrastructure Transport	• Uncooperative motorist refusing to yield to the ambulance • Harassment by social miscreants • Poor road conditions • High traffic density restricted transport • Long transport distance
Cannoodt et al. 2012 [19]	Multiple countries	Systematic review A review of published evidence published in PubMed	General	Not mentioned	Not mentioned	Not mentioned	Not mentioned	Culture/Community Infrastructure Communication/Coordination Transport Equipment Personnel	• Woman relies a males relative for permission • Preference of traditional approach • Lack of knowledge on danger signs • Poor road conditions • Inability to triage patients • Long transport distance • Lack of transportation • Ambulance not available • Lack of equipment • Shortage or lack of training of emergency responders
Nicks et al. 2012 [25]	Tanzania	Evaluation/Report A descriptive overview of the EMS	General	Hospital based dispatch	Layperson First aid	Not mentioned	Ambulance	Infrastructure Communication/Coordination Equipment Personnel	• Poor road conditions • Limited communication between responding team and hospital • Ambulance poorly equipped • No formal training for prehospital providers
Nielsen et al. 2012 [26]	Multiple countries	Cross sectional study Survey conducted of EMS leaders in 13 LMIC in Africa	General	Many different systems	Layperson First Aid EMT Nurse Physician Assistant Physician	Call designated number	Ambulance Personal car Rickshaw	Communication/Coordination Personnel Transport	• Emergency responders communicated with receiving facility part of the time • Lack of uniform dispatch number • Lack of formal training for emergency responders • Transportation using alternative means
Bhopal et al. 2013 [16]	Sierra Leone	Evaluation/report Semi-structured interview based qualitative study and thematic analysis + Retrospective analysis data from health office records	Obstetric emergencies	Hospital based dispatch	Layperson	Walk to central location	Motorbike ambulance	Culture/Community Infrastructure Communication/Coordination	• Lack of knowledge on danger sign • Roads in poor condition • No triaging system
Germa et al. 2013 [37]	Ethiopia	Expert review A historical overview of the development of emergency medicine in Addis Ababa,	General	Private EMS system	First aid	Call designated number	Ambulance Patient means (non-ambulance vehicles)	Communication/Coordination Equipment Personnel	• Lacks a coordinated EMS • Patients are not triaged • Emergency responders don’t communicate with receiving hospital • No coordinated response between emergency responders • Lack of a uniform dispatch number • Ambulance are poorly equipped • Lack of standardized training • Lack of training for emergency responders, physicians and nurses
Joshi et al. 2013 [27]	India	Evaluation/ report Expert review with recommendations	General	EMS with paramedics dispatched	Not mentioned	Call designated number	Ambulance	Communication/Coordination Personnel	• Lack of centralized call center to coordinate emergency response • Emergency responder training not standardized
Radjou et al. 2013 [17]	India	Prospective study Descriptive study of Prehospital care of trauma fatalities	Adult trauma	Not mentioned	Not mentioned	Not mentioned	Ambulance	Infrastructure Communication/Coordination Transport Personnel	• Facility inappropriate to teat patient • Lack of coordination between emergency responders and hospital • Long transport times • Emergency responders lack knowledge of appropriate prehospital care • Emergency responders lack awareness of trauma centers
Tayler-Smith et al. 2013 [4]	Burundi	Cross sectional study Retrospective analysis of data on ambulance records, patient registers and logistics records	General obstetrics	EMS with paramedics dispatched Hospital based dispatch	Nurse	Call designated number	Ambulance	Communication/Coordination Transport	• Need better coordination between dispatcher and ambulance crew • Long transfer times
Wilson et al. 2013 [8]	Multiple countries	Systematic review Systematic review in 10 databases	General obstetrics	Not mentioned	Not mentioned	Not mentioned	Taxi cars Taxi motobikes Hired individual cars	Culture/Community Infrastructure Transport	• Women lack autonomy • Using emergency may summon evil or bad luck • Poor road conditions especially in rainy season • Long wait times and long transport times
Butrick et al. 2014 [34]	Zambia	Cross sectional study Retrospective analysis of data from a randomized cluster trial	Obstetric emergency	EMS with paramedics dispatched	Nurse	Call designated number	Ambulance	Personnel	• Providers lack training to recognize danger signs
Echoka 2014 [23]	Kenya	Cross sectional study Interview based qualitative study and thematic analysis of data from women that experienced obstetric complications	Obstetric emergency	Hospital based dispatch	Nurse Physician	Walk to central location	Ambulance Hired car	Culture/Community Infrastructure Transport Coordination/Communication	• Lack of knowledge on danger signs • Facility inappropriate to treat patient • Road in poor condition • Lack of availability of ambulance
Sidney et al. 2014 [30]	India	Cross sectional study Survey taken of women at qualifying health facilities	Obstetrics	EMS with paramedics dispatched	Not mentioned	Call designated number	Ambulance Personal car Hired car	Culture/Community Infrastructure Transport	• Lack of attendant to accompany patient to the hospital • Poor road condition • Transportation delays • Use of alternative means of transportation • Lack of availability of ambulance • Long transport times
Elbashir et al. 2015 [24]	Sudan	Cross sectional study Qualitative descriptive study based on data from Ministry of Health, internet, interviews and empirical observations	General	EMS with paramedics dispatched	First aid	Call designated number	Ambulance Personal car Taxi cars	Communication/Coordination Transport Personnel	• Emergency response number not well publicized • Lack of structures to help coordinate response to correct location • Long response times • Roads in poor condition • Lack of availability of ambulance • No training for EMS providers or dispatchers
Wesson et al. 2015 [18]	Kenya	Cross sectional study Interview based qualitative study with data from of key informants and focus groups	Adult trauma	Hospital based dispatch	Layperson First aid	Call designated number	Ambulance Personal car Taxi cars	Culture/Community Communication/Coordination Personnel Transport	• Preference of traditional or religious alternatives • No central dispatcher • Poor coordination of the emergency response • Community not aware of emergency number • Emergency number thought by some as not functional • Ambulance unreliable • Lack of trained first responders
Kumar et al. 2009 [52]	India	Cross sectional study Qualitative descriptive study based on observations and structured questionnaire of one participant	General	Hospital based dispatch	Not mentioned	Not mentioned	Ambulance	No EMS system exists	• Skilled personnel

#### Synthesis of the barriers

**Fig. 2:**
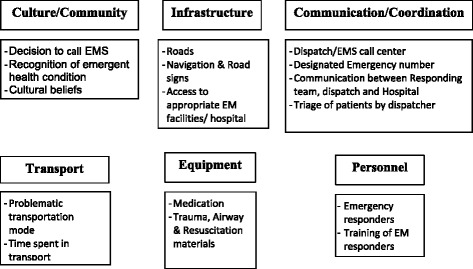
Topics included to define each OHEC barrier

The barrier domains and the EMS pathway barriers were defined a priori [9, 19]. After the data was collected reviewers combined and reorganized these two concepts into the following themes: culture/community, infrastructure, communication/coordination, transport, equipment, and personnel. Like terms and concepts were combined. This new framework simplifies how barriers are viewed, see Fig. 2.

Culture/community represents the knowledge that an individual and/or community may have to recognize an emergent health condition or cultural beliefs that affect the decision to seek urgent medical attention. Infrastructure combines concepts from transportation infrastructure (i.e. road quality, accessible roads and road networks, navigation friendly, etc.) with access to appropriate health facilities (i.e. access to community health center vs. trauma center after a major accident). Communication/coordination represents the ability for a care seeker to call for help through a designated phone number and also for the care provider to be able to coordinate the response effort: this involves effective communication between the responders, the dispatcher, and the hospital. This category also includes the ability for the dispatcher to triage patients appropriately. Transport represents the means that is used to get the patient to an appropriate health facility. Equipment represents the resuscitative equipment and medications necessary to stabilize a patient for transport to a health facility. Lastly, personnel involve the presence and training of personnel responding to emergencies.

#### Assessing risk of bias

Three reviewers (AJ, GK, and PH) assessed study bias using the modified RTI risk of bias tool for observational studies. Articles were assessed using the following domains: inclusion/exclusion criteria, participant recruitment, blinding of study assessors, validity/reliability of study measures, length of study follow-up, loss to follow-up, assessments of harms in study, study limitations and confounding [20]. Each reviewer was assigned a set of articles to review and results were collected using a common abstraction tool.

## Results

### Overview

Of the 1927 unique titles identified in our search strategy, 31 articles were included for full review (Table 2). Most, 77% (24/31), identified three or more categories of barriers present within their respective health systems (Table 3). Primary authors of most of the articles, 55% (17/31), were from outside of the study country (Table 4). Financial constraints at the level of the care-seeker, the institution and the country had significant impacts at multiple levels in the development of OHEC and were not further explored in this review.

**Table 3:** Summary of OHEC barrier discussed by the articles in the systematic review

Barriers Type	Number of articles
Culture/community	39% (12/31)
Infrastructure	55% (17/31)
Communication/coordination	45% (14/31)
Transport	55% (17/31)
Equipment	29% (9/31)
Personnel	61% (19/31)
Number of Barriers mentioned	
6 barriers	6% (2/31)
5 barriers	13% (4/31)
4 barriers	32% (10/31)
3 barriers	26% (8/31)
2 barriers	10% (3/31)
1 barriers	3% (1/31)
0 barriers	13% (4/31)

**Table 4:** Aggregate characteristics of the articles from the review

Study Design	
Observational	71% (22/31)
Reviews	13% (4/31)
Evaluations/reports	13% (4/31)
Qualitative	3% (1/31)
Geographic Focus	
Sub-Saharan Africa	58% (18/31)
India	16% (5/31)
Pakistan	13% (4/31)
More than one country	13% (4/31)
Populations Addressed	
Urban	39% (12/31)
Rural	16% (5/31)
Both	39% (12/31)
Disease Focus	
Medical emergencies	45% (14/31)
Obstetric care	39% (12/31)
Trauma care	16% (5/31)
Type of OHEC system	
Hospital-based	29% (9/31)
Paramedic-based	26% (8/31)
Physician-based	3% (1/31)
Private company	3% (1/31)
No organized OHEC (bystanders or family members provide care)	13% (4/31)
Topic not addressed	26% (8/31)

### Culture/community

Several studies focused on differences in culture and impact on healthcare seeking behavior. For example, in the obstetric literature, it has been reported that once a patient recognizes a need for medical attention, the decision to seek care is not solely their own; decisions to seek care may be influenced by the mother-in-law [3, 21] husband/male relatives [8, 21], or a village elders [11]. For some, more familiar traditional approaches were preferred to newer methods that had not yet gained widespread community approval [12, 14, 18, 21]. Knowledge about and the ability to recognize medical danger signs was another area of focus for many articles [3, 11, 12, 14, 16, 21]. Authors noted that patients were often not aware of when they should seek immediate care.

### Infrastructure

Poor road conditions and poor road networks were the most commonly addressed deficits, 48% (15/31), for transportation infrastructure. Other issues that contribute to this barrier include: sharing roads between the public and emergency vehicles, increased travel distance, lack of road signs, eroded terrains, and narrow roads [2, 8, 11–13, 16, 21–23]. Seasonal difficulties, such as difficulty passing through roads during the rainy season, were commonly reported [3, 8, 12, 13, 16]. In addition to poor infrastructure preventing timely access to care, some articles noted that it also contributed to increased frequency of traffic accidents [2, 22, 24] and increased concerns for transporting patients safely to health facilities [2, 3]. Of the articles that identified an infrastructure barrier, 59% (10/17) noted that patients were first treated by a facility that did not match the required level of care and 47% (8/17) reported that an emergency medical center was not available. Articles also raised concerns about safety and security preventing patients from seeking care at night [21] or impeding emergency responders’ ability to attend to patients [22].

### Communication/coordination

Thirty-nine percent (12/31) described a designated phone number that care seekers could call to activate EMS, and 19% (6/31) specifically mentioned that care seekers had no means of accessing the EMS system. Two papers (6%) noted that patients or their loved ones had to walk to the nearest hospital or clinic to get help. Patients also contacted their local doctors, who didn’t immediately refer them to an appropriate treatment centers [14, 25]. Other barriers that were noted include: lack of awareness of an emergency number, reliability of the number [18, 24], a lack of trust that assistance would show, a lack of public confidence in the EMS [18], and lack of a uniform dispatch number [26]. Additionally, due to unreliable caller information it was difficult to respond to the correct location [24]. Also communication between the ambulance team and the receiving hospital was often limited [17, 25, 26] or uncoordinated [4].

Coordination and management of EMS systems was noted as a barrier in nearly half of articles, 48%, (15/31). Ambulances were often not available when requested [3, 23] or response efforts were duplicated [27]. A quarter of the articles (8/31), identified dispatch/EMS call center as a deficiency in the current EMS system. Deficiencies include, a complete lack or incomplete coverage by the dispatch center, lack of training for the dispatchers, or delays in relaying information [2, 18]. Triage was also identified as a barrier in 19% of articles (6/31), often not being performed at all [16, 28].

### Transport

Most papers, 71% (22/31), reported ambulances as one of the means of transporting patients. However, the use of alternative means such as hired cars (5/31), taxis (8/31), motorcycles (3/31), bicycles (2/31), rickshaws (1/31), public transport buses (5/31), and animal (donkey, horse or ox) drawn carts (4/31) were also present. Among those articles with ambulances present, it was not guaranteed that it was available to transport patients when needed. Lack of fuel, mechanical failure, challenge moving a woman in labor, need to attend to another patient, and prohibitive costs were among the reasons cited for reduced access to ambulances [2, 3, 11, 12, 14, 18, 19, 21, 23, 29, 30]. An Indian study showed that 35% of patients were transported to the hospital via ambulance [15, 31]. Many studies also noted the distance and time it took to get the patient to a hospital being a challenge [2–4, 11, 15, 17, 22, 24, 25, 32]. Time of day also mattered, as transportation at night was not considered safe for fear of being carjacked [18].

### Equipment

Equipment used in a trauma response was identified as a barrier by 36% (5/14) of studies. Hospital ambulances lacked things such as burn kits, spine immobilization boards [29, 32], or the equipment didn’t function [2]. Airway, 21% (3/14), resuscitation equipment, 21%, (3/14), and appropriate medications, 29% (4/14), were also lacking. On some occasions healthcare personnel travelling in the ambulance were asked to carry their own equipment [15].

### Personnel

Lack of formal training was common [2, 24, 26, 29, 32, 33]. More than half of the articles, (18/31), noted that the health system lacked skilled personnel, leaving providers unable to recognize either signs of serious illness [14, 16, 34], the need for a higher level of care [14], or unable to manage acute emergencies [15, 17, 35]. Additionally, some patients were harmed by receipt of inappropriate treatment [15, 17]. In health systems that lacked emergency responders, non-medical personnel like taxi drivers, police, or good Samaritans with little to no training took over the response duties [18, 29, 36]. In one third, (10/31), of articles, OHEC was delivered by laypersons with no training, and 29% (9/31) noted that it was delivered by a person trained in first-aid. Ten percent (3/31) of articles mentioned specifically trained prehospital providers (EMT) being involved with prehospital care (see Table 5). The health system in our review also lacked standards and regulations to guide OHEC services [24, 27].

**Table 5:** Personnel level of training that are discussed in the articles

Level of Training	# Articles that Identify Training Level
Not mentioned	35% (12/31)
Layperson	29% (10/31)
First Aid	29% (10/31)
Nurse	18% (6/31)
Other	15% (5/31)
EMT	12% (4/31)
Doctor	9% (3/31)
Physician Assistant	3% (1/31)

Note: Some articles mentioned more than one of the options listed above

### Risk of Bias

**Fig. 3:**
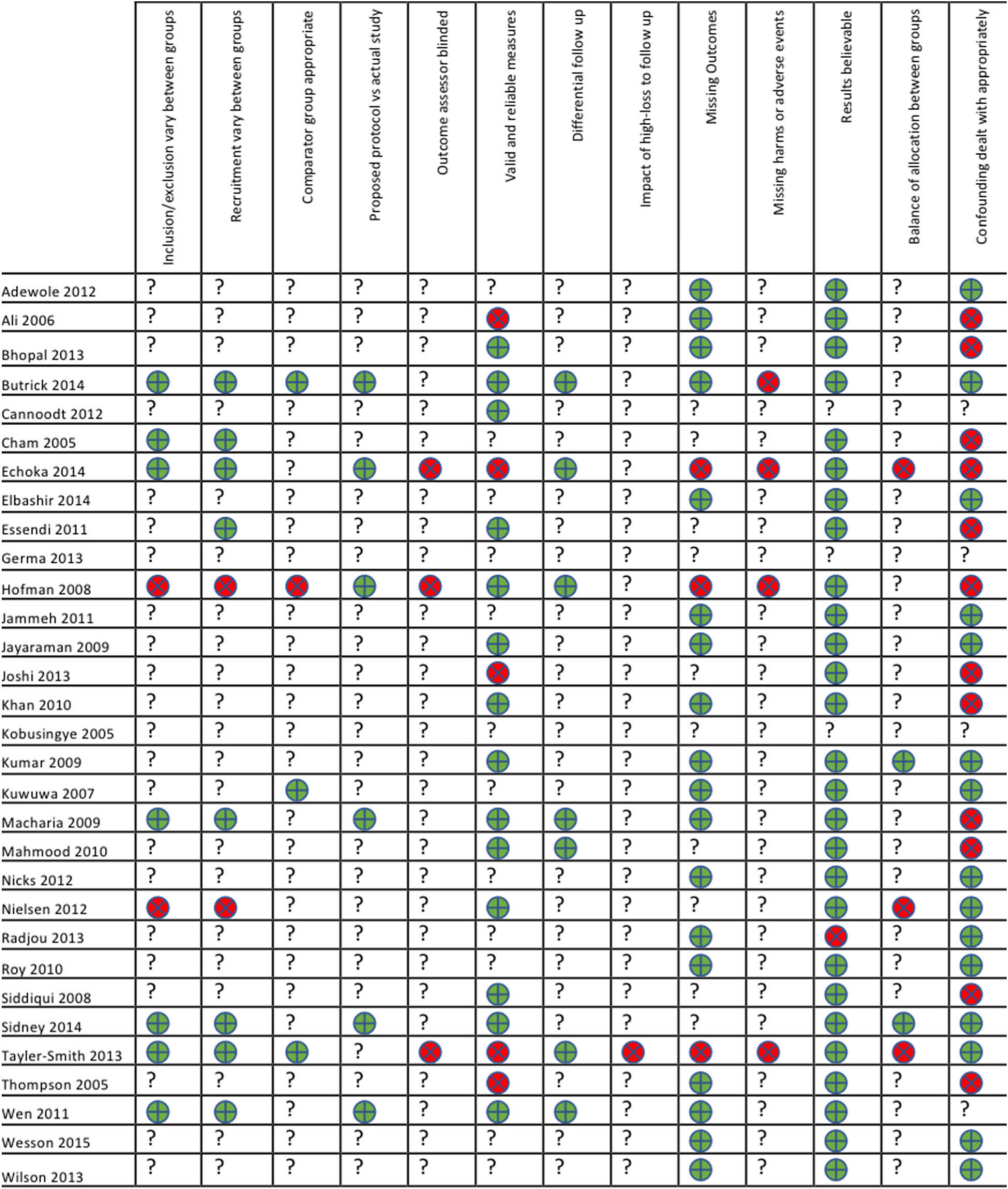
Risk of bias summary. Legend:  Study met criteria.  Study didn’t meet criteria.**?** Not applicable

Of the studies included, six could be evaluated comprehensively using our risk of bias tool [4, 13, 23, 28, 34, 36] and six could not be assessed for bias because they were review articles or evaluation/report article [8, 9, 19, 25, 27, 37]. The remaining articles were studies where most of the domains from our risk of bias tool were not applicable. Overall, most of the studies had believable results (Fig. 3).

## Discussion

This review highlights a lack of standardization in how OHEC was defined and also in how results pertaining to OHEC were reported. Therefore, we adopted concepts from literature to form a framework that divides OHEC into six categories, in a hope to standardize the topics of discussions as we look to improve OHEC in LIC and LMIC.

### Culture/community

Many factors that influence whether an individual will seek care for an emergent health condition are within the locus of the individual and/or community. The main barriers from our review were: lack of knowledge to recognize danger signs, preferences for traditional approaches, and for many women, lack of authority to make ones’ own decisions. Across the literature gender differences in seeking and receiving care are well characterized, particularly in cultures where women have lower levels of literacy and social standing in society than men [38]. While these were the most commonly cited barriers relating to culture and community, they are not necessarily the most important ones. Our understanding of such barriers may be limited by the studies being undertaken by foreign led teams; 56% of the retrieved articles had a primary author from outside of the study country.

Community education has been shown to be successful in addressing community-centered barriers to access [39]. Increased ability to recognize danger signs coupled with awareness and trust of health services can lead to increased utilization of medical services [35] and reductions of delays. Bhopal et al. 2013 used community meetings and film to increase community awareness of new medical services [16]. Increased emphasis on education of women may also help to address gaps in care between men and women. Additionally, working and collaborating with traditional healthcare providers has shown to be beneficial for patients [40]. Moreover, efforts to encourage community members to attend the health facilities for routine and preventative care can also help to familiarize them with the providers and a culture of care delivery [41]. Further research is required, specifically on how community structures can impact service utilization and the barriers that may result.

### Infrastructure

As expected, poor roads that were unreliable - especially during bad weather - played a large role in determining access to care by causing significant delays to patient transport. Further delays also result when patients are taken to facilities that cannot meet their care needs. While many of the barriers in infrastructure noted may be common in rural as well as urban areas, poor roads and scarcity of health facilities are more common in rural areas. However, living in an urban area doesn’t guarantee better access to transport or facility infrastructure, as roads are likely more congested [32] and health centers overcrowded [42, 43].

It is estimated that mortality increases by 2% with every 10% increase in distance traveled to get to a health facility [10]. Thus innovative solutions such a motorcycle transport systems may provide a temporary solution, especially in areas where road quality is not amenable to ambulance transport [13, 16]. Also with navigation affecting transport time [35, 44], innovative solutions that utilize mobile phone and location services may improve time to reaching the patient.

### Communication/coordination

Patients are often very limited in their ability to call for help; 39% of the health systems described (12/31) had a designated emergency phone number. Out of 178 countries surveyed by the WHO, 56 (31%) did not have such numbers [45]. Absence of a uniform number adds an extra hurdle when anxiety and worry are high; having a centralized number is a more efficient means of activating the EMS system [46]. Coordination between the different service providers was also noted to be a formidable challenge. A lack of coordination in a health system where resources are scarce can lead to misallocation of resources or in patients not receiving the appropriate level of care. Multifactorial causes are responsible including: lack of coordination between emergency responders and hospitals, lack of training and limited ability to provide care during long transports and scarcity of higher level care centers.

Coordination can be improved by a centralized EMS system with a regional/local coordinating office. This office would be governed by a committee of representatives from health facilities, transportation authorities, local government administration and community representatives [9] to ensure active participation of all stakeholders. Using this as the backbone of the emergency response system, services like a uniform emergency access telephone number and coordination between ambulance services that currently exist can then function more effectively. Better coordination between parties involved in giving OHEC can lead to improved health outcomes and may also lead to less reliance on expensive technology-intensive models like those of high-income countries [35].

### Transportation

Ambulances were the most commonly cited means of transporting patients, but not the most accessible means of transportation. In one study, less than 1% of the population had access to an ambulance [8]. In high-income countries ambulance transport allows for the emergency responders to quickly get the patient to a treatment center; in LICs and LMICs use of ambulances may not provide the extra benefits of rapid transport, as other motorists are often uncooperative and refuse to give way [22]. However, even when transport is slow, when the ambulance arrives at the hospital the patients are likely attended to more quickly by healthcare providers [34].

In areas with poor road infrastructure, motorbike ambulances have been proposed as better alternatives to vehicles [8, 13, 16]. While motorcycles provide cheaper alternatives for the rapid transportation of patients, they lack in availability of equipment to stabilize and resuscitate patients during transport. Given that motorcycles are significantly cheaper and ubiquitous in many LIC and LMIC, it may be feasible to increase the number of dispatch sites and to supplement current transport with motorcycle ambulances for low acuity patients [13, 18]. Another strategy is to create partnerships between health centers and local transport owners so they too can participate in transporting patients [3, 11].

### Equipment

Lack of equipment prevents emergency responders from giving appropriate care to patients; a Mexican study noted that many patients found in respiratory distress did not get appropriate care due to a lack of equipment [47]. This is in sharp contrast, to the well-equipped ambulances seen in most high-income countries [48]. Furthermore, in LICs and LMICs the personnel knowledge and skill level may not match the equipment available [9].

A list of essential prehospital equipment should be developed; personnel responsible for prehospital care should be trained in the proper utilization of that equipment [49]. A sustainable method of acquiring and replenishing the equipment should be established.

### Personnel

Overwhelmingly, a lack of training for the prehospital providers was identified as a barrier. Few health systems had standards and regulations to guide OHEC services. In settings where emergency responders did not exist, untrained bystanders attempt to give care and even transport patients to health centers. In high-income settings where the health system is more formally organized, bystanders primarily serve the role of activating EMS.

The primary focus of training should be on basic live-saving skills [9]. Training can also help to reduce the risk of secondary injuries sustained during the prehospital period [18]. Additionally, training of essential personnel like taxi drivers and the police in basic life support is recommended [29]. Lastly, laypeople trained in basic first-aid skills have been shown to help improve patient outcomes in several settings [50, 51].

### Finances

As has been illustrated by previous studies, a lack of financial resources is commonly cited as a significant barrier to OHEC development, including both individual financial barriers and system funding. Our review did not go in depth on these barriers. However, a recent systematic review identified six barriers and proposed a cost sharing model as a way to help bridge the gap in OHEC [19] even though the model may put financial pressures on individuals, sharing the cost between the whole community lessens the burden while giving access to important OHEC to all [8, 16]. All other barriers presented in this review must be seen in the context of underpinning financial challenges.

## Limitations

This review is restricted to English peer-reviewed literature and thus sustentative descriptions of OHEC in the grey literature or in policy statements may be overlooked. Additionally, our search did not yield studies that featured OHEC in Latin America, South America, Western Europe or Southeast Asia. This exclusion limits the generalizability of the results. Additionally, while most articles had results that were believable, it was difficult to assess bias given the heterogeneity of the studies.

## Conclusion

Policy makers and researchers seeking to improve OHEC care should focus on improving access, transport, and the availability of trained providers. It is important for leaders in OHEC development to perform feasibility analyses to systematically review deficits in the current OHEC system and the potential solutions to address them. Financial barriers undoubtedly impact resource availability and future efforts to develop tools that can prioritize the components of OHEC most impacting a local community’s ability to deliver OHEC will be central to these efforts.

## Appendix 1

### Search Strategy

#### Pubmed Search

("barrier"[tw] OR "barriers"[tw] OR "delay"[tw] OR "delays"[tw] OR "lack of funding"[tw] OR "lack of communication"[tw] OR "poor roads"[tw] OR "public awareness"[tw] OR "improved access"[tw] OR "insufficient health care"[tw] OR "insufficient healthcare"[tw] OR "lack of equipment"[tw] OR "lack of supplies"[tw] OR "infrastructure"[tw] OR "inadequate funding"[tw] OR "inadequate communication"[tw] OR "inadequate roads"[tw] OR "inadequate access"[tw] OR "inadequate health care"[tw] OR "inadequate healthcare"[tw] OR "inadequate equipment"[tw] OR "inadequate supplies"[tw] OR "lack of training"[tw] OR "inadequate training"[tw] OR "local support"[tw] OR "health care access"[tw] OR "healthcare access"[tw] OR "lack of resources"[tw] OR "inadequate resources"[tw]) AND

("Emergency Medical Services"[Mh:noexp] OR "emergency medical services" OR "emergency health service" OR "EMS" OR "emergency medical service" OR "Advanced Trauma Life Support Care"[Mesh] OR "advanced trauma life support" OR "Emergency Medical Service Communication Systems"[Mesh] OR "emergency medical service communication systems" OR "Transportation of Patients"[Mesh] OR "transportation of patients" OR "patient transportation" OR "patient transport" OR "Ambulances"[Mesh] OR "ambulances" OR "ambulance" OR "emergency mobile unit" Or "emergency mobile units" OR "Air Ambulances"[Mesh] OR "air ambulances" OR "air medical transport" OR "emergency helicopter" OR "emergency helicopter" OR "life flight" OR "Triage"[Mesh] OR "triage" OR "prehospital" OR "pre-hospital" OR "EMT" OR "emergency medical technician" OR "emergency medical technicians" OR "paramedic" OR "paramedics" OR "dispatcher" OR "dispatch")

AND

### Combine: (#1 OR #2 OR #3 OR #4 OR #5 OR #6 OR #7)

**#1 Search** "developing country"[tiab] OR "developing countries"[tiab] OR "developing nation"[tiab] OR "developing nations"[tiab] OR "developing population"[tiab] OR "developing populations"[tiab] OR "developing world"[tiab] OR "less developed country"[tiab] OR "less developed countries"[tiab] OR "less developed nation"[tiab] OR "less developed nations"[tiab] OR "less developed population"[tiab] OR "less developed populations"[tiab] OR "less developed world"[tiab] OR "lesser developed country"[tiab] OR "lesser developed countries"[tiab] OR "lesser developed nation"[tiab] OR "lesser developed nations"[tiab] OR "lesser developed population"[tiab] OR "lesser developed populations"[tiab] OR "lesser developed world"[tiab] OR "under developed country"[tiab] OR "under developed countries"[tiab] OR "under developed nation"[tiab] OR "under developed nations"[tiab] OR "under developed population"[tiab] OR "under developed populations"[tiab] OR "under developed world"[tiab] OR "underdeveloped country"[tiab] OR "underdeveloped countries"[tiab] OR "underdeveloped nation"[tiab] OR "underdeveloped nations"[tiab] OR "underdeveloped population"[tiab] OR "underdeveloped populations"[tiab] OR "underdeveloped world"[tiab] OR "middle income country"[tiab] OR "middle income countries"[tiab] OR "middle income nation"[tiab] OR "middle income nations"[tiab] OR "middle income population"[tiab] OR "middle income populations"[tiab] OR "low income country"[tiab] OR "low income countries"[tiab] OR "low income nation"[tiab] OR "low income nations"[tiab] OR "low income population"[tiab] OR "low income populations"[tiab] OR "lower income country"[tiab] OR "lower income countries"[tiab] OR "lower income nation"[tiab] OR "lower income nations"[tiab] OR "lower income population"[tiab] OR "lower income populations"[tiab] OR "underserved country"[tiab] OR "underserved countries"[tiab] OR "underserved nation"[tiab] OR "underserved nations"[tiab] OR "underserved population"[tiab] OR "underserved populations"[tiab] OR "underserved world"[tiab] OR "under served country"[tiab] OR "under served countries"[tiab] OR "under served nation"[tiab] OR "under served nations"[tiab] OR "under served population"[tiab] OR "under served populations"[tiab] OR "under served world"[tiab] OR "deprived country"[tiab] OR "deprived countries"[tiab] OR "deprived nation"[tiab] OR "deprived nations"[tiab] OR "deprived population"[tiab] OR "deprived populations"[tiab] OR "deprived world"[tiab]

**#2 Search** "poor country"[tiab] OR "poor countries"[tiab] OR "poor nation"[tiab] OR "poor nations"[tiab] OR "poor population"[tiab] OR "poor populations"[tiab] OR "poor world"[tiab] OR "poorer country"[tiab] OR "poorer countries"[tiab] OR "poorer nation"[tiab] OR "poorer nations"[tiab] OR "poorer population"[tiab] OR "poorer populations"[tiab] OR "poorer world"[tiab] OR "developing economy"[tiab] OR "developing economies"[tiab] OR "less developed economy"[tiab] OR "less developed economies"[tiab] OR "lesser developed economy"[tiab] OR "lesser developed economies"[tiab] OR "under developed economy"[tiab] OR "under developed economies"[tiab] OR "underdeveloped economy"[tiab] OR "underdeveloped economies"[tiab] OR "middle income economy"[tiab] OR "middle income economies"[tiab] OR "low income economy"[tiab] OR "low income economies"[tiab] OR "lower income economy"[tiab] OR "lower income economies"[tiab] OR "low gdp"[tiab] OR "low gnp"[tiab] OR "low gross domestic"[tiab] OR "low gross national"[tiab] OR "lower gdp"[tiab] OR "lower gnp"[tiab] OR "lower gross domestic"[tiab] OR "lower gross national"[tiab] OR lmic[tiab] OR lmics[tiab] OR "third world"[tiab] OR "lami country"[tiab] OR "lami countries"[tiab] OR "transitional country"[tiab] OR "transitional countries"[tiab]

**#3 Search** Africa[tiab] OR Asia[tiab] OR Caribbean[tiab] OR West Indies[tiab] OR South America[tiab] OR Latin America[tiab] OR Central America[tiab] OR "Atlantic Islands"[tiab] OR "Commonwealth of Independent States"[tiab] OR "Pacific Islands"[tiab] OR "Indian Ocean Islands"[tiab] OR "Eastern Europe"[tiab] OR Afghanistan[tiab] OR Albania[tiab] OR Algeria[tiab] OR Angola[tiab] OR Antigua[tiab] OR Barbuda[tiab] OR Argentina[tiab] OR Armenia[tiab] OR Armenian[tiab] OR Aruba[tiab] OR Azerbaijan[tiab] OR Bahrain[tiab] OR Bangladesh[tiab] OR Barbados[tiab] OR Benin[tiab] OR Byelarus[tiab] OR Byelorussian[tiab] OR Belarus[tiab] OR Belorussian[tiab] OR Belorussia[tiab] OR Belize[tiab] OR Bhutan[tiab] OR Bolivia[tiab] OR Bosnia[tiab] OR Herzegovina[tiab] OR Hercegovina[tiab] OR Botswana[tiab] OR Brasil[tiab] OR Brazil[tiab] OR Bulgaria[tiab] OR Burkina Faso[tiab] OR Burkina Fasso[tiab] OR Upper Volta[tiab] OR Burundi[tiab] OR Urundi[tiab] OR Cambodia[tiab] OR Khmer Republic[tiab] OR Kampuchea[tiab] OR Cameroon[tiab] OR Cameroons[tiab] OR Cameron[tiab] OR Camerons[tiab] OR Cape Verde[tiab] OR Central African Republic[tiab] OR Chad[tiab] OR Chile[tiab] OR China[tiab] OR Colombia[tiab] OR Comoros[tiab] OR Comoro Islands[tiab] OR Comores[tiab] OR Mayotte[tiab] OR Congo[tiab] OR Zaire[tiab] OR Costa Rica[tiab] OR Cote d'Ivoire[tiab] OR Ivory Coast[tiab] OR Croatia[tiab] OR Cuba[tiab] OR Cyprus[tiab] OR Czechoslovakia[tiab] OR Czech Republic[tiab] OR Slovakia[tiab] OR Slovak Republic[tiab] OR Djibouti[tiab] OR French Somaliland[tiab] OR Dominica[tiab] OR Dominican Republic[tiab] OR East Timor[tiab] OR East Timur[tiab] OR Timor Leste[tiab] OR Ecuador[tiab] OR Egypt[tiab] OR United Arab Republic[tiab] OR El Salvador[tiab] OR Eritrea[tiab] OR Estonia[tiab] OR Ethiopia[tiab] OR Fiji[tiab] OR Gabon[tiab] OR Gabonese Republic[tiab] OR Gambia[tiab] OR Gaza[tiab] OR Georgia Republic[tiab] OR Georgian Republic[tiab] OR Ghana[tiab] OR Gold Coast[tiab] OR Greece[tiab] OR Grenada[tiab] OR Guatemala[tiab] OR Guinea[tiab] OR Guam[tiab] OR Guiana[tiab] OR Guyana[tiab] OR Haiti[tiab] OR Honduras[tiab] OR Hungary[tiab] OR India[tiab] OR Maldives[tiab] OR Indonesia[tiab] OR Iran[tiab] OR Iraq[tiab] OR Isle of Man[tiab] OR Jamaica[tiab] OR Jordan[tiab] OR Kazakhstan[tiab] OR Kazakh[tiab] OR Kenya[tiab] OR Kiribati[tiab] OR Korea[tiab] OR Kosovo[tiab] OR Kyrgyzstan[tiab] OR Kirghizia[tiab] OR Kyrgyz Republic[tiab] OR Kirghiz[tiab] OR Kirgizstan[tiab] OR "Lao PDR"[tiab] OR Laos[tiab] OR Latvia[tiab] OR Lebanon[tiab] OR Lesotho[tiab] OR Basutoland[tiab] OR Liberia[tiab] OR Libya[tiab] OR Lithuania[tiab]

**#4 Search** Macedonia[tiab] OR Madagascar[tiab] OR Malagasy Republic[tiab] OR Malaysia[tiab] OR Malaya[tiab] OR Malay[tiab] OR Sabah[tiab] OR Sarawak[tiab] OR Malawi[tiab] OR Nyasaland[tiab] OR Mali[tiab] OR Malta[tiab] OR Marshall Islands[tiab] OR Mauritania[tiab] OR Mauritius[tiab] OR Agalega Islands[tiab] OR "Melanesia"[tiab] OR Mexico[tiab] OR Micronesia[tiab] OR Middle East[tiab] OR Moldova[tiab] OR Moldovia[tiab] OR Moldovian[tiab] OR Mongolia[tiab] OR Montenegro[tiab] OR Morocco[tiab] OR Ifni[tiab] OR Mozambique[tiab] OR Myanmar[tiab] OR Myanma[tiab] OR Burma[tiab] OR Namibia[tiab] OR Nepal[tiab] OR Netherlands Antilles[tiab] OR New Caledonia[tiab] OR Nicaragua[tiab] OR Niger[tiab] OR Nigeria[tiab] OR Northern Mariana Islands[tiab] OR Oman[tiab] OR Muscat[tiab] OR Pakistan[tiab] OR Palau[tiab] OR Palestine[tiab] OR Panama[tiab] OR Paraguay[tiab] OR Peru[tiab] OR Philippines[tiab] OR Philipines[tiab] OR Phillipines[tiab] OR Phillippines[tiab] OR Poland[tiab] OR Portugal[tiab] OR Puerto Rico[tiab] OR Romania[tiab] OR Rumania[tiab] OR Roumania[tiab] OR Russia[tiab] OR Russian[tiab] OR Rwanda[tiab] OR Ruanda[tiab] OR Saint Kitts[tiab] OR St Kitts[tiab] OR Nevis[tiab] OR Saint Lucia[tiab] OR St Lucia[tiab] OR Saint Vincent[tiab] OR St Vincent[tiab] OR Grenadines[tiab] OR Samoa[tiab] OR Samoan Islands[tiab] OR Navigator Island[tiab] OR Navigator Islands[tiab] OR Sao Tome[tiab] OR Saudi Arabia[tiab] OR Senegal[tiab] OR Serbia[tiab] OR Montenegro[tiab] OR Seychelles[tiab] OR Sierra Leone[tiab] OR Slovenia[tiab] OR Sri Lanka[tiab] OR Ceylon[tiab] OR Solomon Islands[tiab] OR Somalia[tiab] OR Sudan[tiab] OR Suriname[tiab] OR Surinam[tiab] OR Swaziland[tiab] OR Syria[tiab] OR Syrian[tiab] OR Tajikistan[tiab] OR Tadzhikistan[tiab] OR Tadjikistan[tiab] OR Tadzhik[tiab] OR Tanzania[tiab] OR Thailand[tiab] OR Togo[tiab] OR Togolese Republic[tiab] OR Tonga[tiab] OR Trinidad[tiab] OR Tobago[tiab] OR Tunisia[tiab] OR Turkey[tiab] OR Turkmenistan[tiab] OR Turkmen[tiab] OR Tuvalu[tiab] OR Uganda[tiab] OR Ukraine[tiab] OR Uruguay[tiab] OR USSR[tiab] OR Soviet Union[tiab] OR Union of Soviet Socialist Republics[tiab] OR Uzbekistan[tiab] OR Uzbek OR Vanuatu[tiab] OR New Hebrides[tiab] OR Venezuela[tiab] OR Vietnam[tiab] OR Viet Nam[tiab] OR West Bank[tiab] OR Yemen[tiab] OR Yugoslavia[tiab] OR Zambia[tiab] OR Zimbabwe[tiab] OR Rhodesia[tiab]

**#5 Search** Developing Countries[Mesh] OR Africa[Mesh:NoExp] OR Africa, Northern[Mesh:NoExp] OR Africa South of the Sahara[Mesh:NoExp] OR Africa, Central[Mesh:NoExp] OR Africa, Eastern[Mesh:NoExp] OR Africa, Southern[Mesh:NoExp] OR Africa, Western[Mesh:NoExp] OR Asia[Mesh:NoExp] OR Asia, Central[Mesh:NoExp] OR Asia, Southeastern[Mesh:NoExp] OR Asia, Western[Mesh:NoExp] OR Caribbean Region[Mesh:NoExp] OR West Indies[Mesh:NoExp] OR South America[Mesh:NoExp] OR Latin America[Mesh:NoExp] OR Central America[Mesh:NoExp] OR "Atlantic Islands"[Mesh:NoExp] OR "Commonwealth of Independent States"[Mesh:NoExp] OR "Pacific Islands"[Mesh:NoExp] OR "Indian Ocean Islands"[Mesh:NoExp] OR "Europe, Eastern"[Mesh:NoExp]

**#6 Search** Afghanistan[Mesh] OR Albania[Mesh] OR Algeria[Mesh] OR American Samoa[Mesh] OR Angola[Mesh] OR "Antigua and Barbuda"[Mesh] OR Argentina[Mesh] OR Armenia[Mesh] OR Azerbaijan[Mesh] OR Bahrain[Mesh] OR "Baltic States"[Mesh] OR Bangladesh[Mesh] OR Barbados[Mesh] OR Benin[Mesh] OR "Republic of Belarus"[Mesh] OR Belize[Mesh] OR Bhutan[Mesh] OR Bolivia[Mesh] OR Bosnia-Herzegovina[Mesh] OR Botswana[Mesh] OR Brazil[Mesh] OR Bulgaria[Mesh] OR Burkina Faso[Mesh] OR Burundi[Mesh] OR Cambodia[Mesh] OR Cameroon[Mesh] OR Cape Verde[Mesh] OR Central African Republic[Mesh] OR Chad[Mesh] OR Chile[Mesh] OR China[Mesh] OR Colombia[Mesh] OR Comoros[Mesh] OR Congo[Mesh] OR Costa Rica[Mesh] OR Cote d'Ivoire[Mesh] OR Croatia[Mesh] OR Cuba[Mesh] OR Cyprus[Mesh] OR Czechoslovakia[Mesh] OR Czech Republic[Mesh] OR Slovakia[Mesh] OR Djibouti[Mesh] OR "Democratic Republic of the Congo"[Mesh] OR "Democratic People's Republic of Korea"[Mesh] OR Dominica[Mesh] OR Dominican Republic[Mesh] OR East Timor[Mesh] OR Ecuador[Mesh] OR Egypt[Mesh] OR El Salvador[Mesh] OR Eritrea[Mesh] OR Estonia[Mesh] OR Ethiopia[Mesh] OR "Equatorial Guinea"[Mesh] OR Fiji[Mesh] OR "French Guiana"[Mesh] OR Gabon[Mesh] OR Gambia[Mesh] OR "Georgia (Republic)"[Mesh] OR Ghana[Mesh] OR Greece[Mesh] OR Grenada[Mesh] OR Guatemala[Mesh] OR Guinea[Mesh] OR Guinea-Bissau[Mesh] OR Guam[Mesh] OR Guyana[Mesh] OR Haiti[Mesh] OR Honduras[Mesh] OR Hungary[Mesh] OR "Independent State of Samoa"[Mesh] OR India[Mesh] OR Indonesia[Mesh] OR Iran[Mesh] OR Iraq[Mesh] OR Jamaica[Mesh] OR Jordan[Mesh] OR Kazakhstan[Mesh] OR Kenya[Mesh] OR Korea[Mesh] OR Kyrgyzstan[Mesh] OR Laos[Mesh] OR Latvia[Mesh] OR Lebanon[Mesh] OR Lesotho[Mesh] OR Liberia[Mesh] OR Libya[Mesh] OR Lithuania[Mesh] OR "Macedonia (Republic)"[Mesh] OR Madagascar[Mesh]

**#7 Search** Malawi[Mesh] OR Malaysia[Mesh] OR Mali[Mesh] OR Malta[Mesh] OR Mauritania[Mesh] OR Mauritius[Mesh] OR "Melanesia"[Mesh] OR Mexico[Mesh] OR Micronesia[Mesh] OR Middle East[Mesh:NoExp] OR Moldova[Mesh] OR Mongolia[Mesh] OR Montenegro[Mesh] OR Morocco[Mesh] OR Mozambique[Mesh] OR Myanmar[Mesh] OR Namibia[Mesh] OR Nepal[Mesh] OR Netherlands Antilles[Mesh] OR New Caledonia[Mesh] OR Nicaragua[Mesh] OR Niger[Mesh] OR Nigeria[Mesh] OR Oman[Mesh] OR Pakistan[Mesh] OR Palau[Mesh] OR Panama[Mesh] OR Papua New Guinea[Mesh] OR Paraguay[Mesh] OR Peru[Mesh] OR Philippines[Mesh] OR Poland[Mesh] OR Portugal[Mesh] OR Puerto Rico[Mesh] OR "Republic of Korea"[Mesh] OR Romania[Mesh] OR Russia[Mesh] OR "Russia (Pre-1917)"[Mesh] OR Rwanda[Mesh] OR "Saint Kitts and Nevis"[Mesh] OR Saint Lucia[Mesh] OR "Saint Vincent and the Grenadines"[Mesh] OR Samoa[Mesh] OR Saudi Arabia[Mesh] OR Senegal[Mesh] OR Serbia[Mesh] OR Montenegro[Mesh] OR Seychelles[Mesh] OR Sierra Leone[Mesh] OR Slovenia[Mesh] OR Sri Lanka[Mesh] OR Somalia[Mesh] OR South Africa[Mesh] OR Sudan[Mesh] OR Suriname[Mesh] OR Swaziland[Mesh] OR Syria[Mesh] OR Tajikistan[Mesh] OR Tanzania[Mesh] OR Thailand[Mesh] OR Togo[Mesh] OR Tonga[Mesh] OR "Trinidad and Tobago"[Mesh] OR Tunisia[Mesh] OR Turkey[Mesh] OR Turkmenistan[Mesh] OR Uganda[Mesh] OR Ukraine[Mesh] OR Uruguay[Mesh] OR USSR[Mesh] OR Uzbekistan[Mesh] OR Vanuatu[Mesh] OR Venezuela[Mesh] OR Vietnam[Mesh] OR Yemen[Mesh] OR Yugoslavia[Mesh] OR Zambia[Mesh] OR Zimbabwe[Mesh]

## Abbreviations

AFEM: African Federation for Emergency Medicine
EMS: Emergency medical services
FRC: First responder care
LIC: Low-income countries
LMIC: Low-middle income countries
OHEC: Out of hospital emergency care
PHC: Prehospital care
WHO: World Health Organization
